# Imagining Coat‐Hangers and Pills: A Qualitative Exploration of Abortion Beliefs and Attitudes in Hostile Policy Contexts in the United States

**DOI:** 10.1111/psrh.12289

**Published:** 2025-01-09

**Authors:** Rosalyn Schroeder, Lori R. Freedman, Andréa Becker, Chris Ahlbach, M. Antonia Biggs

**Affiliations:** ^1^ Department of Obstetrics, Gynecology & Reproductive Sciences University of California San Francisco California USA

**Keywords:** abortion, abortion attitudes, abortion knowledge, medication abortion, self‐managed abortion

## Abstract

**Objective:**

We explored awareness of and attitudes about the safety of various methods people use to attempt to end a pregnancy without medical assistance, which we refer to in this study collectively as self‐managed abortion (SMA).

**Methods:**

In 2020, we invited individuals living in eight United States (US) states considered “hostile” to abortion rights or with a history of criminalizing abortions performed outside the formal healthcare system to participate in semi‐structured telephone interviews regarding their attitudes toward these practices. We analyzed coded transcripts for content and themes.

**Results:**

We interviewed 54 individuals. Participants perceived methods of ending a pregnancy on one's own to have a high potential for complications, often evoking “coat hanger” abortions. Participants also frequently referenced methods such as physical trauma, herbs, teas, alcohol, or other drugs. Very few participants reported awareness of medication abortion pills. When asked about the safety of SMA in the context of self‐sourcing these medications, participants considered pills safer and more acceptable than other SMA methods, while still fearing incorrect use and complications. Others believed that SMA could offer greater reproductive autonomy, less stigma, and a safer physical and psychological experience than facility‐based abortion care.

**Conclusion:**

In 2020, most participants perceived SMA as involving unsafe practices and did not include use of medication abortion pills. Future research should document how beliefs and attitudes have been influenced by the expansion in telemedicine provision of medication abortion, the implementation of new state abortion bans, and the promulgation of Shield Laws.

## 1 Introduction

In 2017, researchers estimated that approximately 7% of women in the United States (US) will to try to end an unwanted pregnancy on their own during their lifetime, using methods such as blunt force, herbs, alcohol, drugs, and medication abortion pills [1]. Following the *Dobbs v Jackson Women's Health Organization (Dobbs)* decision in 2022 that overturned *Roe v. Wade* (*Roe*), a 1973 US Supreme Court case that established a federal constitutional right to an abortion [2], requests for and use of medication abortion pills purchased outside the formal US health system increased markedly [3, 4]. In the face of legal and logistical obstacles and diminished access to facility‐based abortion care [5, 6], paired with the increased availability of medication abortion from online distributers [7, 8], the number of individuals considering and/or attempting to self‐manage an abortion with medication abortion pills or other, potentially unsafe, methods is increasing, becoming the only feasible pathway to abortion for some [9, 10, 11].

In this study, we refer to the various ways to attempt to end one's pregnancy outside the formal US healthcare system as self‐managed abortion (SMA) [12]. In the US context, SMA (historically referred to as self‐induced abortion) may involve the use of a range of methods that vary in their safety and effectiveness, including physical interventions, such as placing foreign objects into the body or being hit in the abdomen; use of herbs, illicit drugs, alcohol, or medications not intended to end a pregnancy; or self‐sourcing the abortion medication(s) misoprostol (with or without mifepristone) from the Internet or other sources [13, 14].

According to recent US studies, women, transgender men, and gender non‐binary individuals with the capacity for pregnancy may choose to end a pregnancy on their own due to barriers in accessing facility‐based care, concerns about privacy or for greater reproductive autonomy, considerations around physical or emotional safety, or to avoid the stigma they might experience seeking in‐person abortion care. Abortion seekers may also opt to end a pregnancy on their own because they perceive it to be more affordable, more expedient, more convenient, or to provide a more person‐centered experience than clinical care may offer [5, 15, 16, 17, 18, 19, 20].

While the practice of ending a pregnancy on one's own has been common throughout history [21], most popular narratives regarding these practices in the US posit it as unsafe, which may in part be reflective of the widespread sharing of cautionary tales relating to morbidities and mortalities resulting from unsafe pre‐*Roe* abortions [22, 23] and the ubiquitousness of the “coat hanger abortion” in common parlance [24]. In addition, longstanding stigma associated with abortion has led to a dearth of accurate knowledge about abortion and the range of SMA practices [25, 26], including inaccurate and over‐sensationalized media portrayals about the dangers of abortion and SMA [27, 28]. However, research examining abortion seekers' experiences ending a pregnancy on their own outside the formal healthcare system in the US found that few experienced complications requiring medical intervention [1] and that they often prioritized methods that were safe and available, although not necessarily effective [18].

In the US, there are new and rapidly expanding options available for individuals to self‐source medication abortion pills from organizations and online pharmacies that provide access to these medications outside the purview of the formal US healthcare system [4, 7]. In areas where abortion is legally restricted or banned, people have for decades relied on medication abortion—often via misoprostol procured through local pharmacies or social networks—as a preferred method to safely and effectively end a pregnancy outside the formal healthcare system [29, 30, 31]. Accordingly, the World Health Organization has recently outlined in its 2022 global abortion guidelines how the practice of self‐managing an abortion with misoprostol with or without mifepristone medications can be a “potentially empowering and active extension of the health system” [32].

In the US, the lifting of the US Food and Drug Administration (FDA)'s in‐person dispensing requirement for mifepristone [33] has allowed for expanded access to and use of safe and effective mail‐order medication abortion options through telemedicine [34, 35, 36]. While in this study we refer to the self‐sourcing and use of medication abortion pills accessed outside the formal US healthcare system (e.g., by ordering pills online from an international organization) as self‐managed medication abortion,^1^ this practice may largely appear similar to accessing medication abortion pills through a telemedicine service. With telemedicine, a licensed healthcare provider consults with a patient via a remote video, phone, or chat messaging service (synchronous) or through an asynchronous online platform to determine a patient's medical eligibility and mails medication abortion pills to the patient's preferred location; this practice can often resemble the experience of self‐sourcing of medication abortion pills via online distributors working outside the formal US healthcare system, who may also connect patients with resources that offer virtual, yet informal, medical, emotional, or legal support services [8, 12, 37].

While the process of telemedicine and SMA with medication abortion can be similar, women, transgender men, and gender non‐binary individuals who self‐manage an abortion may face additional legal threats [12, 37, 38, 39]. Currently 14 US states ban abortion in almost all circumstances, and at least 26 states, including some states considered as protective of access to abortion, have historically attempted to misapply other existing statutes to criminalize people suspected of attempting to end their own pregnancy between 2000 and 2020 [40]. While some of these regulations have been enacted under the pretext that they improve public health safety, efforts to criminalize SMA have been denounced by leading public health and medical organizations [41, 42, 43]. Legal advocates now suggest that the greatest risk to a person, particularly women of color and those living on low incomes, who self‐manages an abortion is not from the abortion itself but from the threat of legal punishment for doing so [44, 45, 46].

While most people in the US believe that abortions performed in a clinical setting are safe [47], little research has explored people's attitudes regarding the safety of various methods of ending a pregnancy on one's own without clinician assistance [48]. In the current sociopolitical moment occurring post‐*Roe*, during which SMA attempts are predicted to increase [4, 13], it is critical to understand lay perceptions of the safety of SMA because exaggerated danger in the popular imagination may increase the criminalization and reporting of vulnerable people.

In this paper, we explore attitudes related to SMA among individuals who at the time of the study in 2020 were living in US states considered “hostile” or “very hostile” to abortion^2^ [50]; many of these states have also enacted laws banning SMA directly or have a history of misapplying state criminal laws to motivate the investigation and/or prosecution of individuals suspected of attempting to end a pregnancy on their own [40, 51]. We sought to understand better if and how views of individuals living in these abortion‐hostile policy environments reflect the policies enacted in these states. Throughout this manuscript, we consider all methods to attempt to end a pregnancy that our participants could recall or imagine as forms of SMA, since at the time of this study mail‐order dispensing of medication abortion was prohibited in the US.

## 2 Methods

### 2.1 Sample and Recruitment

We recruited individuals to participate in telephone interviews from February to December 2020 via Facebook advertisements. All potential participants completed an online eligibility survey through Qualtrics that collected data on the prospective participant's age, state of residence at the time of the study, sex at birth, gender identity, sexual identity, race and ethnicity, highest level of education completed, and religion. Those assigned female at birth were additionally asked about their birth parity and their history of abortion.

To be eligible to participate in the study, individuals needed to be at least 18 years old, speak English, have access to a telephone, and live in one of eight states identified as hostile to abortion rights (Arkansas, Idaho, Indiana, Louisiana, Mississippi, Missouri, South Carolina, and South Dakota) [50].

While state of residence was our primary sampling characteristic, we also aimed to recruit participants who represented a broad range of demographic characteristics, including gender, age, race/ethnicity, and religion. When possible, we attempted to balance for 2020 US Census demographic characteristics in our purposive sample. While we included religion as a component of the purposive sampling frame to capture diverse perspectives, given that religious beliefs are often tied to perceptions of abortion legality and morality in the US [52, 53], religious identity and religiosity were rarely mentioned by the participants, and thus we do not address it in the results.

### 2.2 Gender Inclusiveness

We conducted our interviews in two phases, stratified by sex assigned at birth. In the first phase, we interviewed 25 adults (aged 18–65) assigned male at birth (AMAB), all of whom identified as cisgender men. These respondents lived in one of the six states categorized as “very hostile” to abortion rights at the time of the interviews, which included Arkansas, Indiana, Louisiana, Mississippi, Missouri, and South Dakota [50]. This first set of interviews focused on broad views about abortion, including SMA.

In the second phase, we interviewed 29 adults (aged 18–45) assigned female at birth (AFAB), which included 27 cisgender women, one respondent who identified as nonbinary, and one who identified as a transgender man. In this second phase, we recruited from the same states as the first phase and added Idaho and South Carolina because they had laws in place that specifically allow for the criminal investigation or arrest of individuals suspected of ending a pregnancy on their own [51]. In this second phase, we included additional probes specific to SMA.

### 2.3 Data Collection

RS, a woman with masters degrees in public health and demography and one co‐author (CA), a male medical student researcher, conducted all interviews by telephone. Both interviewers are trained in qualitative research methods and in‐depth interviewing techniques.

We designed interview guides to be semi‐structured, which allowed participants to introduce new ideas while also ensuring that certain topics were covered. We began interviews with a series of questions about interviewees' general and community attitudes toward abortion and access to abortion services. We then explored participants' personal reproductive health experiences, including their history of pregnancy and abortion. Using language that has been cognitively tested and reported in the published literature [1, 54], we elicited participants' knowledge of abortion methods someone might use to “end a pregnancy on their own without medical assistance” as well as their perceptions regarding the safety of ending a pregnancy on one's own more broadly. We also inquired about participants' awareness of medication abortion, their views about its safety, and their thoughts on the legality of and/or criminalization of using it when self‐sourced outside of the formal US healthcare system. Interviews averaged 73 min (men's: 89 min; women's: 58 min), and we offered participants a USD50 electronic Amazon gift card to thank them for their time.

### 2.4 Data Analysis

We audio‐recorded all interviews and transcribed audio files using a professional transcription service. We reviewed transcripts for accuracy prior to data analysis. Interviewers then coded and analyzed interviews in Dedoose through multiple iterations; we first identified a preliminary set of broad themes and then tracked a more nuanced set of themes and subthemes. The authorship team met to discuss codes and we refined the final codebook after multiple iterations of thematic organization and distillation of codes. RS and CA reviewed and independently coded three identical transcripts before meeting to discuss their individual coding strategies to identify and correct any inconsistencies in coding practices. After coming to consensus, RS and CA divided the remaining transcripts and coded each independently while periodically reviewing together to confirm alignment in ongoing coding practices. After we applied codes to all transcripts, the full authorship team conducted a thorough thematic analysis within and between codes to investigate emergent themes found in the interview data. The authorship team met biweekly during data analysis and coding processes to discuss ongoing findings, which informed our interpretations of the data.

For this paper, we analyzed transcripts and interview memos for content and themes related to interviewees' familiarity with and views on the safety of different methods someone might use to end a pregnancy on their own, including the use of medication abortion pills obtained outside of the formal US healthcare system. We organized results by three domains of inquiry: (1) awareness of SMA methods; (2) views of safety by method; and (3) perceptions of risks and opportunities of self‐managed medication abortion. We have published on themes related to these same participants' general perceptions about SMA abortion legality and criminality previously [55].

### 2.5 Ethical Considerations

Western Institutional Review Board determined our study to be minimal risk and exempt from review. All participants first consented to participate in the study on an electronic eligibility survey and then provided oral consent for audio‐recording at the beginning of the interview. We assigned each participant a pseudonym and removed all personally‐identifying information.

## 3 Results

### 3.1 Participant Characteristics

From a total of 605 individuals that completed a preliminary eligibility and demographic survey, 514 people screened as eligible. We purposively selected 54 participants for age, gender, race/ethnicity, religion, and geographic area to participate in semi‐structured telephone interviews, in an attempt to balance for 2020 US Census demographic estimates. As such, the demographic characteristics of our participants reflected that of their states of residence with the majority self‐identifying as white and cisgender [56]. Participants ranged in age from 18 to 48 (average: 32 years). We present the sample characteristics in Table 1.

**TABLE 1:** Characteristics of interview participants from eight abortion hostile states in the United States (*N* = 54).

Characteristic	Number (%)
Age	
18–24	11 (20%)
25–29	10 (19%)
30–34	10 (19%)
35–39	13 (24%)
40–44	6 (11%)
45 or older	4 (7%)
Gender	
Woman	27 (50%)
Man	25 (46%)
Non‐binary	1 (2%)
Trans man	1 (2%)
State of residence	
Arkansas	7 (13%)
Idaho	5 (9%)
Indiana	12 (22%)
Louisiana	11 (20%)
Mississippi	6 (11%)
Missouri	5 (9%)
South Carolina	5 (9%)
South Dakota	3 (6%)
Race/ethnicity	
Asian	2 (4%)
Native American/American Indian	2 (4%)
Black/African American	11 (20%)
Mixed race	4 (7%)
White	32 (59%)
Other	3 (6%)
Identifies as Hispanic or Latinx	
Yes	10 (19%)
No	42 (78%)
Missing	2 (4%)
Religion	
Atheist, Agnostic, or “None”	18 (33%)
Baptist	8 (15%)
Buddhist	1 (2%)
Catholic	9 (17%)
Christian other (includes Evangelicals)	13 (24%)
Jewish	1 (2%)
Muslim	2 (4%)
*Questions below were asked only of people who were assigned female at birth* (*n* = 29)	
Number of Children	
0	11 (38%)
1	6 (21%)
2	6 (21%)
3–5	3 (10%)
More than 5	2 (7%)
Has had an abortion	
Yes	8 (28%)
No	19 (66%)
Not sure	2 (7%)

*Note:* Data in this table were collected as part of the eligibility survey for the study.

### 3.2 Participant Awareness of Methods to End a Pregnancy on One's Own

Early in the interview, we prompted participants to think about “people who end their pregnancy without the help of a medical provider” and identify different methods they had heard about people using to do so. Most participants mentioned one or more methods, with the “coat hanger” or “back alley” abortion being reported most frequently. Despite not knowing anyone who had used this method, it was the most enduring narrative mentioned, which cut across generational and gender groups. Participants also reported widespread awareness of herbs, teas, drugs, or medicines not specifically designed to induce an abortion being taken to end a pregnancy. Others had heard of people using physical trauma, including intentionally falling down the stairs or being punched or kicked in the stomach, to attempt to end a pregnancy. Ryan (cisgender man, age 30), from Missouri, identified as morally opposed to abortion yet also believed that it should remain legal, summarized many SMA methods, while also acknowledging the “coat hanger” abortion as a stereotype:

Obviously, there's the stereotype of using the coat hanger. And I've heard about people drinking things, whether they make their own sort of concoction or they drink something that is even poisonous or harmful to their adult bodies and tended to be overwhelmingly poisonous to the fetus.

Multiple participants had heard personal stories of loved ones or acquaintances attempting to end a pregnancy on their own. One respondent, Amber (cisgender woman, age 32), from Arkansas, who had two previous abortions yet had mixed feelings about other people seeking multiple abortions, disclosed having considered using physical trauma to end a pregnancy that ultimately ended in an in‐clinic abortion:

At the time [of my pregnancy] I was panicking, not really knowing what to do. Like can I just fall down the stairs? And I'm like, no, I'll end up breaking my neck and then, just being paralyzed and pregnant at the same time. It was never anything like a serious thing, where I thought about sticking a coat hanger up there or anything like you see in the movies and the TV shows. It was never anything serious like that. But the thought definitely came across.

Few participants (12 women and 3 men) had ever heard of medication abortion in general, and no one knew of anyone who had considered self‐sourcing medication abortion pills from the Internet or another resource. Participants assigned female at birth had higher awareness of various types of methods to end one own's pregnancy than men (see Table 2).

**TABLE 2:** Self‐managed abortion methods mentioned by interview participants from eight abortion hostile states in the United States (*N* = 54), by method type and sex.

Self‐managed abortion method(s) mentioned	No. of women, transmen, and gender non‐binary individuals^a^ reporting awareness (*n* = 29)	No. of cisgender men reporting awareness (*n* = 25)	Total (*N* = 54)
Inserting items into the body (including “coat hanger”, “hanger”, and “hook”)	22	7	29
Use of herbs, teas, alcohol consumption, over‐the counter medications, illicit drug use, chemicals	21	5	26
Physical trauma (includes “stairs,” “violence,” “punch,” “hit,” “kicked”)	12	3	15
Medication abortion pills (includes “misoprostol,” “abortion pills”)	3	0	0

^a^ Includes people assigned female at birth: 27 identified as women, 1 as a transgender man, and 1 as non‐binary.

### 3.3 Views on the Safety of Self‐Managing an Abortion Depended on Method

While most participants believed that all SMA methods were intrinsically unsafe, people tended to be most concerned about the safety of methods that involved physical injury, trauma, or “coat hanger” abortions. They perceived these methods to have the highest risk for causing serious injury or even death. Neil (cisgender man, age 40), from Louisiana and who was supportive of legal access to abortion, shared how the idea of a “coat hanger” abortion was first described to him:

The use of a coat hanger or the term “coat hanger abortion” was something that was kind of brandished about. And I remember thinking like what in the hell is that? And then someone describing it as “this is what it's like, these are what abortions are like.” It's someone going into a seedy place in the city…this is something that bad people do.

Participants also held concerns about the safety of attempting to end a pregnancy via binge‐drinking alcohol or by taking over the counter or prescription drugs. As Ryan stated: “I've even heard of people terminating pregnancies by drinking excessive amounts of alcohol or taking drugs in the hopes that will end their pregnancy…Those are way riskier and worse than all other options, right?”

While some individuals were also concerned by the use of herbs or teas to attempt to end a pregnancy, others presumed these methods to be less effective and less dangerous, while still others saw them as a more natural alternative to clinic‐based care. Jasmine (cisgender woman, age 27), from Indiana and supportive of legal abortion access, explained: “I started doing more research onto what happens or what the [abortion] procedures were before colonialism and discovered that women had been doing this for a really long time with massages and with herbs and botanicals – and like roots and barks and things from the forest, all natural.”

### 3.4 Perceptions of Risk and Opportunity of Self‐Sourced Medication Abortion

After describing how people typically access procedural and medication abortion in a clinic, we asked participants about their thoughts regarding the safety of using medication abortion accessed outside of the formal healthcare system: “While some people may get their abortion pills from a clinic, other people may get these same abortion pills by ordering them online. Do you think it would be safe for someone to take this medication without the help of a medical provider? Why or why not?”

When prompted to think about the safety of self‐sourced medication abortion pills, participants—and particularly those who were unaware of medication abortion or who were unsupportive of abortion access in general—expressed concerns that the medications ordered online might be fraudulent, unsafe, or ineffective. Hikari (cisgender woman, age 23), from Louisiana and who held personal moral apprehensions about abortion but believed it should be legal, noted: “I don't think it's safe. They need to take it under a medical provider's supervision…do we actually trust that those [online medications] are actually the ingredients for an abortion as opposed to getting it medically prescribed by your medical provider who knows about the prescription and whatever else comes along with it?”

Many participants felt uneasy about the use of medication abortion without clinical supervision, describing concerns about excessive bleeding or infection that may lead to future infertility or even death. As Mira (cisgender woman, age 19), from Arkansas and who identified as “pro‐abortion” and “pro‐choice 100%” described: “I don't remember what the other side effects were, but I know excessive bleeding could be an issue. I mean, that would be even if you're taking the right dose. Say somebody orders the wrong dosage for their body weight and height off the Internet, it could cause them a lot of harm. I know infertility could be an issue.”

Meredith (cisgender woman, age 35), from Louisiana and who believed all abortions should be illegal without any exceptions, was aware that complications relating to abortion were rare, yet this further motivated her to believe that when abortions do go “wrong” they are especially dangerous. She pointed to abortion medications' lack of over‐the‐counter approval as evidence that it would be unsafe to self‐source medication abortion pills.

Complications—although rare—can be severe…I believe a woman has a right to quality healthcare and that includes making sure that the side effects aren't going to be contraindicative to their unique medical condition. The idea behind over‐the‐counter meds is they are supposed to be so safe that they would not, in the general population, cause adverse reactions. I don't think that's true of the abortion pill.

While Kaya (cisgender woman, age 32), from Idaho, was supportive of legal abortion access, she was leery about people self‐sourcing medication abortion pills due to negative experiences she had using misoprostol to treat her previous miscarriages. She warned: “From my own experience, I've had missed miscarriages and I've taken Cytotec or misoprostol, and it did not work and I've gotten infections, so knowing that could happen to someone else even if they do get the real thing online…you can end up with serious complications, infections, and whatnot.” Ada (cisgender woman, age 36), from South Carolina, had previously had an in‐clinic abortion and was supportive of legal abortion access but perceived attempts to self‐manage an abortion via medication abortion pills as intrinsically dangerous: *“*I think it's always safer to use a medical professional. Go to the doctor, let them tell you that [medication abortion] pills are okay to take. I think people are trying to kill themselves if they ever try to terminate their pregnancy by themselves.”

Most respondents reported at least some reservations about using medication abortion pills ordered online, yet many—particularly those who identified as supportive of abortion rights more generally—still perceived this to be a safer and more acceptable method as compared to other alternatives. Brooklyn (cisgender woman, age 29), from Indiana and supportive of legal abortion access, believed that medication abortion offered a lower potential for risk than other methods: “[I've heard] some really scary, bad stuff, you know? Clothes hanger abortions…having somebody else kick their stomach until they were no longer pregnant. I've heard of people swallowing bleach and other chemicals…I think it's a case where if an abortion pill becomes available, I think that would make it safe.”

Faizel (cisgender man, age 26), from Mississippi and who was supportive of legal abortion access, noted that a pill regimen would provide a simpler and safer option for those considering ending a pregnancy on their own: “I feel like the medical knowledge required for, ‘Here's a pill…that has a lot less room for error than ‘fall down a flight of stairs’ or ‘take this wire hanger.’’.”

Other participants who expressed support for abortion rights were more confident about the safety of using self‐sourced medication abortion pills. Jasmine described: “I think it [medication abortion] is probably as safe as birth control [pills]—which, again, causes problems for people and is perfectly safe for millions of others.” Finally, Jonathan (cisgender man, age 22), from Indiana believed that access to abortion should remain legal, as he perceived the self‐sourcing of medication abortion pills online as a form of harm reduction: “I'd support it to just give people a more readily available option to perform a safe abortion, as opposed to try and take things into their own hands, doing it in illegal, potentially unsafe ways.”

Still, most participants believed that medication abortion should ideally be accessed within the framework of a medical model to ensure ongoing access to medical expertise and intervention if needed, which respondents perceived would lead to fewer complications. Alexis feared a bad outcome otherwise: “When you think of an at‐home abortion, the first thing that really comes into your mind is pretty gruesome, it's pretty gnarly. If we can prevent that, that's a good thing…People die, there's a risk of infection, there's just so many things that could possibly go wrong in that scenario without a medical professional.”

Despite concerns about the safety of self‐sourcing medication abortion pills and the assertion that clinician involvement was preferrable, many participants viewed the idea of being able to order medication abortion pills online as providing an opportunity for more person‐centered abortion care. Brooklyn explained: “I feel like if a woman wants to choose to do that in the privacy of her own home, where she feels comfortable and feels safe, then I feel like they should be given the choice and option to do so.” Mira believed that this option would reduce barriers to abortion care for those whose healthcare access may otherwise be marginalized: “I definitely think not having to get a prescription [when accessing abortion pills online] would be an extremely crucial aspect to it because a lot of women who really need abortions are ones that don't have a lot of money. A lot of times, of course, that ties into poverty and lack of healthcare options.”

Others saw expanding online access to medication abortion as a way to reduce abortion stigma and protect patient privacy. Lisa (cisgender woman, age 38) from South Carolina who had previously experienced harassment from anti‐abortion protesters when she attended an in‐clinic medication abortion appointment, explained that ordering pills online would be a more private and less stigmatizing option to end a pregnancy: “Sometimes, there are people judging you and stuff, and it's probably just easier to do it at home.” Trinity (cisgender woman, age 18), from Idaho who was supportive of legal abortion access, agreed: “Ordering [pills] online would definitely help with some of the stigma I believe…even buying them in store or having to go into a clinic, especially with some of those protesters outside of a clinic that you have to kind of deal with…If I was ever in that situation, it would make me more comfortable to have that [mail order] option.”

Others similarly saw the opportunity to avoid the increased physical and psychological stress posed by protesters outside of abortion clinics. Felicia (cisgender woman, age 32), from South Carolina, could still recall her stressful experience with abortion protesters more than a decade after her in‐clinic procedure and believed that self‐sourcing medication abortion pills could provide an opportunity to prevent these harms for others:

When I had my abortion at 19, I had to walk through an actual protest line to get an abortion. That was very traumatic for me, I wish hell on all those people because it was already a traumatic experience for me to make a decision to have an abortion…it's a very traumatic experience when you have somebody that is against abortion and looks like they want to kill you because you're making a decision about your body. If you didn't want to experience someone actually protesting and making you feel bad about this, then, yeah, by all means. Confidentiality. Get the pill sent to your house and you go through that, that's cool too.

With a greater emphasis on physical safety, Zo, a 35‐year‐old transgender man from Louisiana who was highly supportive of abortion and reported knowing several family members who had successfully ended their pregnancies using herbal teas and other ancestral remedies, also saw the option of self‐sourcing medication abortion pills online to self‐manage one's own abortion experience as offering a way to bypass clinic protesters: “I mean it's not any less safe than going into a clinic that has a person who's like threatening to kill you. It's kind of the same risk,” and he summed up abortion clinic protesters as “*homegrown terrorism*.” Zo also described his interest in self‐sourcing medication abortion pills as a risk‐preventative measure due to a personal mistrust of formal healthcare facilities as a Black and Latinx person that had felt marginalized in these spaces in the past:

When you go into any kind of [healthcare] facility, you immediately feel kind of shame or you get, you know, afraid…I think that a lot of people are okay with the idea of just kind of relying on the medical field, and it's kind of not really realizing that if you don't look a certain way, you won't be respected in that medical field. And so Black and Brown people go to doctors all the time and they just don't trust the doctors. And it's a real thing because those true stories are very, very, very impactful to the other generations, even the ones who didn't experience it.

Zo suggests that SMA via medication abortion pills allows for the opportunity to reduce externalized threats and judgments and perceived this option to be both physically and emotionally safer than seeking in‐person abortion services.

## 4 Discussion

In this study, we found that people living in states with policies that were hostile to abortion prior to *Dobbs* were largely unfamiliar with medication abortion yet were familiar with many other, different types of methods people might use to end a pregnancy without clinical supervision, ranging from physical trauma to using herbs. While all methods to end a pregnancy on one's own were perceived as largely unsafe and ineffective, those caused via physical injury or trauma were considered among the most dangerous and were also the most frequently mentioned. Despite its rarity in our current context, we note the prevailing dominance of the “coat hanger” abortion narrative in the public imagination [18, 57], possibly a result of pervasive dated and dangerous abortion imagery and a dearth of accurate abortion representation in popular media [27].

Participants believed that using herbs to self‐manage an abortion was largely ineffective yet also thought it was relatively benign and more natural, consistent with the limited contemporary literature examining the use of herbs to induce an abortion [21]. While there is a long history of using herbs to end a pregnancy [58, 59], more research is needed to assess their safety and effectiveness for use in SMA [9, 21].

While medication abortion is the most common form of abortion occurring within the formal US healthcare system [60], few people were aware of or mentioned medication abortion as a form of SMA. The low awareness of SMA with medication abortion is consistent with studies indicating its relatively low prevalence among those who have attempted to self‐manage an abortion in the US [57, 61], although this practice may be increasing [3, 4]. We found that many participants indicated support for online telemedicine models for accessing medication abortion, which while available today, were largely unavailable at the time of the study. However, many participants perceived abortion pills self‐sourced from the Internet to be unsafe, noting concerns that the pills may be inauthentic, people might take the pills incorrectly, or that people would be ill‐equipped to manage side effects or potential severe complications. These concerns conflict with evidence demonstrating that medication abortion pills available from online sources are largely authentic and dosed correctly [62], and medication abortion obtained via telemedicine models is very safe and effective and has very low risk of serious complications [63, 64, 65]. Furthermore, research suggests that in a simulated environment, people are able to understand medication abortion label instructions [66] and self‐screen for eligibility without clinical supervision, although more research is needed [67].

As all participants lived in states with laws that are openly hostile to abortion rights [50], their views may also reflect anti‐abortion attitudes and stigma present within their communities, as well as exposure to pervasive misinformation claiming that abortion is physically and emotionally risky [68, 69, 70, 71]. Fears about the safety of abortion when self‐sourcing the medications may be further propagated by state laws that limit abortion access and stigmatize or endorse harmful myths about abortion [69], including telemedicine abortion bans and mandated abortion counseling practices that misinform patients about the risks of abortion [72]. In addition, popular media's portrayal of abortion—including depictions of self‐managed medication abortion—have often been presented as stigmatized, clandestine, and intrinsically unsafe [28, 73], despite few people experiencing serious abortion‐related complications in our current context [74, 75].

Conversely, some participants viewed SMA as a way to exercise reproductive autonomy, to maintain privacy, and to avoid engaging with the formal healthcare system, which can oftentimes be burdensome, stigmatizing, or discriminatory—particularly for abortion seekers living on low‐incomes and women of color [76, 77]. Participants also believed that SMA, particularly when self‐sourcing the medication abortion pills, could provide both increased psychological and physical safety for people living in places that highly stigmatize abortion and where people risk being harassed and/or threatened by abortion clinic protesters when seeking in‐person abortion care [26, 78]. Some participants also viewed self‐sourced medication abortion as a form of harm reduction that they perceived as safer and more reliable than other, alternative methods, views that have similarly been reported among women in Latin America [30]. Moreover, we found that participants who were supportive of legalized abortion access—and were more knowledgeable about abortion in general—were more likely to view abortion, including self‐managed medication abortion, as safe and morally acceptable.

### 4.1 Limitations

Qualitative methods allowed us to explore interview participants' personal views about the safety and availability of methods to end a pregnancy on one's own, which has largely been missing from the literature on this topic. However, we conducted all interviews between February–December 2020. Since this time, there have been major expansions in the availability and use of telemedicine medication abortion services in the US following the FDA's removal of the in‐person mifepristone dispensing requirements and the promulgation of Shield Laws that may impact the applicability of some of the findings presented in this paper to the current moment, as telemedicine medication abortion was largely an unrealized opportunity at the time of the study. These policy changes, in combination with *Dobbs* and the subsequent state abortion bans that followed, have led to immense social change since our data were collected. As such, this study can best be understood as offering baseline qualitative insights about how abortion attitudes may have evolved since 2020, given the massive changes in abortion policy and practice that have occurred since.

We identified and included a diverse set of voices, which we attempted to balance by gender, age, race/ethnicity, socioeconomic status, and religion, within a purposively‐built sample. That said, small samples such as these are best used for describing the range of experiences and perspectives within a defined subset (here: people living in hostile abortion policy contexts) and are not necessarily generalizable or transferable to residents of abortion‐supportive policy contexts or the greater public at large. Our findings intentionally pertain to people living in abortion‐hostile policy contexts in acknowledgment of the increased risk of criminalization of SMA there and in order to assess how heightened stigma and abortion‐hostility may show up in people's beliefs and attitudes generally.

It is also worth qualifying that since participants self‐selected into the study and were eager to speak about abortion, they may have been more interested in the topic of abortion and sympathetic to the need for it—including methods to end a pregnancy on one's own—than others in their area or among the US general public. We suggest that this only makes findings about abortion misinformation and lack of awareness of medication abortion among our sample even more striking.

Finally, the field does not have consistent and widely accepted terminology to distinguish between SMA methods that are known to be dangerous (e.g., physical trauma) and methods that have proven to be very safe and effective (e.g., misoprostol with or without mifepristone) [79]. The rapidly changing policy environment and variability in language used among experts, coupled with widespread misinformation and confusion about abortion, may present a significant barrier to both measuring and informing the general public about self‐managed medication abortion.

## 5 Conclusion

We found that participants had knowledge of a wide variety of different methods a person might use to end a pregnancy on their own, although much of this knowledge leaned on common cultural tropes, such as the “coat hanger” abortion [24]. Despite over 20 years of FDA approval in the US, most interview participants were largely unaware of the existence of medication abortion pills in general and of its potential use to self‐manage an abortion. Once informed about medication abortion, many participants expressed more favorable attitudes to it as an alternative to other methods of ending a pregnancy on one's own, although misperceptions about the method were common and represent major challenges to normalizing its potential use for SMA. Increased public awareness about the existence, safety, and effectiveness of medication abortion pills remains necessary to dispel harmful myths about the method, particularly as attempts to self‐manage an abortion are likely to continue to increase. Future research should document how people's beliefs and attitudes have been influenced by the expansion in telemedicine provision of medication abortion and by the implementation of new state abortion bans.

## Endnotes

^1^
At the time of our interviews in February–December 2020, the use of telemedicine for medication abortion was rarely available, as US FDA regulations prohibited the mail‐order dispensing of mifepristone. This requirement was permanently lifted in December 2021, a move spurred on largely by impacts that the COVID‐19 pandemic had on abortion access across the US. As such, all discussions and quotations throughout this manuscript referring to ordering mail‐order medication abortion pills would have been occurring outside the US formal healthcare system at the time and would be what we call “self‐managed medication abortion.” Since the time of our data collection, major innovations in medication abortion provision have occurred, which have dramatically changed the landscape for medication abortion access via telemedicine.

^2^
We collected our data in 2020. As of the end of 2024, seven states in our eligibility list (Arkansas, Idaho, Indiana, Louisiana, Mississippi, Missouri, and South Dakota) have banned abortion in almost all circumstances, while South Carolina bans abortion at 6 weeks or later [49].
